# Accelerating diversity, equity, and inclusion goals: a qualitative assessment from the lens of scientists at the 2020 Clinical Translational Science Awards Annual Meeting

**DOI:** 10.1017/cts.2022.516

**Published:** 2022-12-07

**Authors:** Claudia S.P. Fernandez, Monica M. Taylor, Gaurav Dave, Kathleen Brandert, Katherine Mollenkopf, Suzanna Larkin, Giselle Corbie

**Affiliations:** 1 Gillings School of Global Public Health, The University of North Carolina, Chapel Hill, NC, USA; 2 Center for Health Equity Research, School of Medicine, The University of North Carolina, Chapel Hill, NC, USA; 3 Department of Medicine, School of Medicine, The University of North Carolina, Chapel Hill, NC, USA; 4 Abacus Evaluation, School of Medicine, The University of North Carolina, Chapel Hill, NC, USA; 5 College of Public Health, University of Nebraska Medical Center, Omaha, NE, USA

**Keywords:** Evaluation metrics, racism, structural change, institutional culture, underrepresented minorities

## Abstract

Participants in the leadership breakout session at the Clinical Translational Science Awards (CTSA) virtual 2020 conference discussed and ranked six recommendations in terms of feasibility, impact, and priority for advancing Diversity, Equity, and Inclusion (DEI) efforts to elevate underrepresented populations to leadership positions in CTSAs and their broader institutions. A thematic analysis of chat and polling data identified challenges and opportunities to achieve DEI goals, with the three most promising recommendations as: cross-institutional Principal Investigator (P.I.) action-learning workgroups, transparent policies for recruiting and promoting underrepresented minorities (URM) leadership, and a clear succession plan to nurture and elevate URM leaders. Suggestions are made to improve DEI in CTSA leadership and allow for greater representation in the translational science field.

## Introduction

As translational science and research incubators, Clinical Translational Science Awards (CTSA) aim to transform the research enterprise through team science, community collaborations, and catalyzing clinical and translational science innovations to meet the needs of communities and patients. Similar to education [1], healthcare [2], and public health [3], CTSAs face challenges in forming effective and equitable community–academic partnerships, with concerns that opportunities for advancing science and practice to alleviate health disparities could be lost [4]. Today, the USA is experiencing stark health inequities perpetuating morbidity and mortality rates and the economic burden [5]. CTSA’s ability to achieve equitable representation in the workforce (Diversity) [6], eliminating barriers to success and productivity (Equity) [7], and creating a culture where all thrive and participate (Inclusion) [7], often referred to as “DEI,” comes into focus as a crucial objective. To implement DEI objectives, a combination of self-awareness, skills, and a commitment from public health and healthcare leaders is needed to change the status quo [8]. Understanding the felt needs and workplace experiences of CTSA scientists and research staff can help inform how organizations can be more inclusive within the structure and interprofessional practices to speed the translation of new science from discovery to application.

**Fig. 1.**
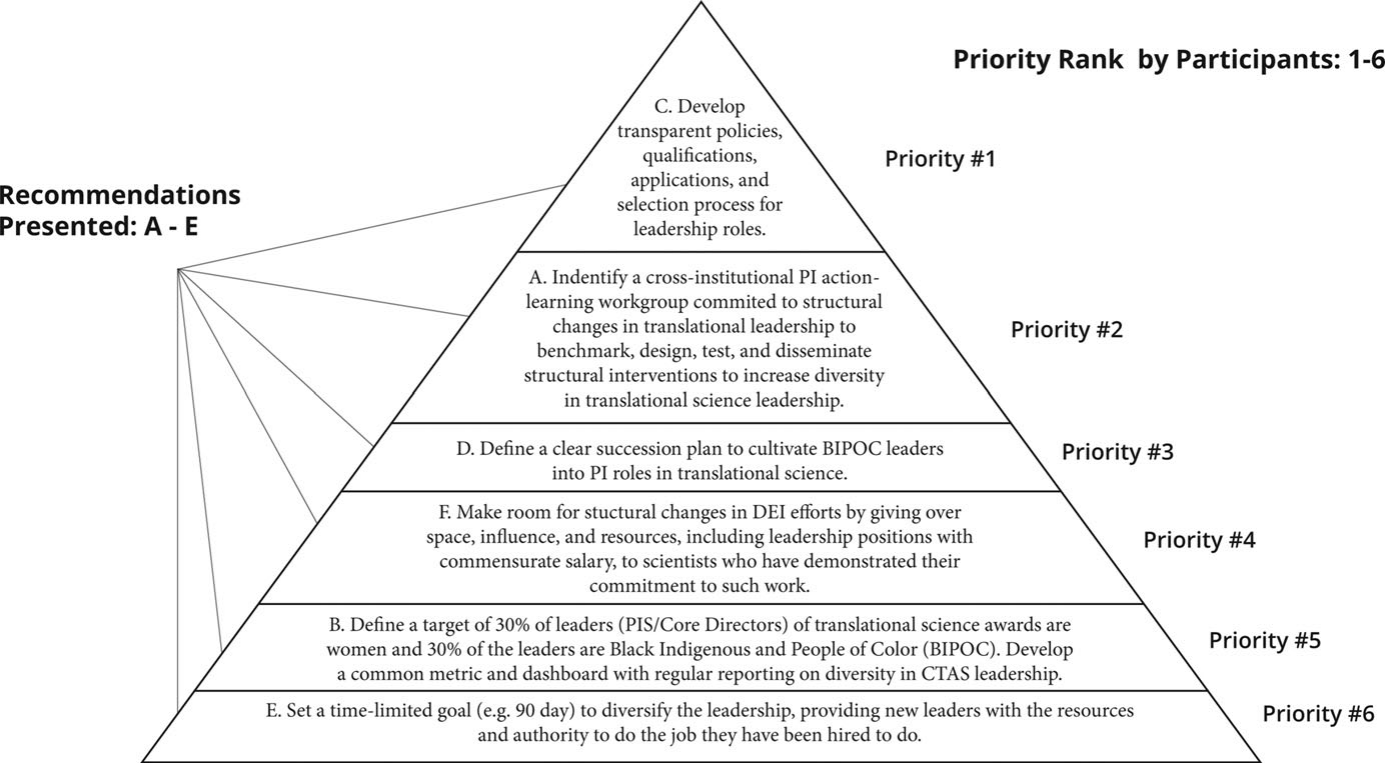
**Priority ranking of six recommendations presented.** Recommendation C (“Develop transparent policies, qualifications, applications and selection process for leadership roles”) = Priority #1; Recommendation A (“Identify a cross institutional P.I. action-learning workgroup committed to structural change in translational leadership to benchmark, design, test, and disseminate structural interventions to increase diversity in translational science leadership”)= Priority #2; Recommendation D (“Define a clear succession plan to cultivate BIPOC leaders into P.I. roles in translational science”)= Priority #3; Recommendation F (“Make room for structural change in DEI efforts by giving over space, influence, and resources - including leadership positions with commensurate salary - to scientists who have demonstrated their commitment to such work”)= Priority #4; Recommendation B (“Define a target of at least 30% of leaders (P.I.s/Core Directors) of translational science awards are women and 30% of the leaders are Black Indigenous and People of Color (BIPOC). Develop a common metric and dashboard with regular reporting on diversity in CTSA leadership”)= Priority #5; Recommendation E (“Set a time limited goal (e.g. 90 days) to diversify the leadership, providing new leaders with the resources and authority to do the job they have been hired to do”)= Priority #6.

In 2020, the national CTSA meeting was a virtual conference on the Zoom videoconferencing application, during which participants were invited to attend “Town Hall” sessions on equity, diversity, and inclusion in clinical and translational research in four key areas: clinical trials recruitment, workforce development, health equity/disparities research, and leadership diversity. The leadership diversity session presented six recommendations (labeled as A–F), developed by research scientists, for advancing DEI and elevating URM leaders in CTSAs and their institutions. The recommendations were based on the literature and professional experiences with equity initiatives and leadership development [8,9]. Six issues were commented on by session participants and then ranked in terms of *feasibility*, defined as the degree to which something could be accomplished in the current environment, *impact*, defined as the capacity to have a meaningful and sustainable paradigm shift over time, and *priority*, defined as the things you would put in place first [10]. Participants then commented on the six defined issues presented by responding to prompted questions in the Zoom chat. Participant comments were analyzed to understand the challenges experienced from the perspective of those in the CTSA workforce, revealing a depth of concerns. We analyzed polling data, visualized as a pyramid model (Fig. 1), ranking the six issues presented (Table 1). This manuscript presents a thematic analysis of the stakeholder input, revealing a preference for cross-institutional P.I. action-learning workgroups, transparent policies for recruiting and promoting leadership in URM, and a clear succession plan to nurture and elevate URM leaders. This manuscript aims to provide feasible suggestions to improve DEI in CTSA leadership and allow for greater representation in the field.

**Table 1.** Response to six recommendations by leadership breakout group participants

Priority 1: Recommendation C: Develop transparent policies, qualifications, applications, and selection process for leadership roles.		
“Theme” Tally “n” (% responses) Total *N* = 44	Definition	Example Quote
“Institutional Culture” 4 (9.09%)	The culture, politics, and administrative structure of institutions that could serve as barriers to DEI expansion in CTSAs	“3A. Very feasibility; limited only by how far the culture has been moved”
“Institutional Buy-in” 5 (11.36%)	Institutional and cultural acceptance and backing of DEI efforts	“3a will take enormous buy-in from all levels of leadership to change. Is feasible but not easy.”
“Evaluation and Monitoring” 7 (15.91%)	The importance of strategies to monitor and evaluate new and ongoing DEI priorities	“3b. Must evaluate the outcomes into actionable items”
Priority 2: Recommendation A: Identify a cross institutional P.I. action-learning workgroup committed to structural change in translational leadership to benchmark, design, test, and disseminate structural interventions to increase diversity in translational science leadership.		
Theme Tally “n” (% responses) Total *N* = 45	Definition	Example Quote
“Cross-Institutional” 5 (11.11%)	Geographic, cultural, political, etc. differences between CTSAs and the institutions in which they operate	“1A. Barrier: CTSA is a cross-university and cross health system activity and each of those have their own culture so often need different variations to succeed”
“New Perspectives” 8 (17.78%)	Expanding DEI in CTSA leadership could result in new, broader, more diverse perspectives	“1B: With more diversity in leadership, overall diversity and priorities may change/improve”
“Expansion” 9 (20.00%)	Expanding diversity efforts beyond the P.I. level to include broader leadership	“1A: I would hope that the aim is not to prepare people to be CTSA PI. I would think we want to enable people to be what they aspire to be/ Leading a CTSA or being a Dean is a low bar. 1A”
Priority 3: Recommendation D: Define a clear succession plan to cultivate BIPOC leaders into P.I. roles in translational science.		
“Theme” Tally “n” (% responses) Total *N* = 47	Definition	Example Quote
“Succession” 12 (25.53%)	The process of developing individuals to take on positions of greater power	“4A. Succession planning and nurturing is always challenging since new opportunities may arise for those being nurtured, however if leadership training is part of this process, then the end result may still add more BIPOC individuals in leadership positions.”
“Resentment” 7 (14.89%)	Non-BIPOC individuals and/or individuals in power may resent pushes to change leadership structures and increase DEI	“4B: How does one remove current individuals who are in a positions, without inflaming bias against the new person of color in this position”
“New Perspectives” 8 (17.02%)	Expanding DEI in CTSA leadership could result in new, broader, more diverse perspectives	“4A. New energy and new ideas. Seeing leadership diversity grows possibilities for all members of the community”

Priority 4: Recommendation F: Make room for structural change in DEI efforts by giving over space, influence, and resources - including leadership positions with commensurate salary - to scientists who have demonstrated their commitment to such work.		
“Theme” Tally “n” (% responses) Total *N* = 44)	Definition	Example Quote
“New Perspectives” 5 (11.36%)	Expanding DEI in CTSA leadership could result in new, broader, more diverse perspectives	“6A ..like the idea- new centers, institutes, initiatives, goals, breadth of work”
“Feasible Implementation” 5 (11.36%)	The degree to which DEI efforts would be feasible at CTSAs and their broader institutions	“6A This may be difficult to implement at institutions where space is under high demand and there are competing priorities”
“Stepping Down” 5 (11.36%)	Those who step down or “give up” their positions as part of DEI expansion efforts and the controversy surrounding that	“6A: The framing of ‘giving over’ or ‘giving up’ is already one of the barriers to change. There’s already a perception ‘qualified’ people are being asked to step aside for ‘unqualified’ people”
Priority 5: Recommendation B: Define a target of at least 30% of leaders (P.I.s/Core Directors) of translational science awards are women and 30% of the leaders are Black Indigenous and People of Color (BIPOC). Develop a common metric and dashboard with regular reporting on diversity in CTSA leadership.		
“Theme” Tally “n” (% responses) Total *N* = 49	Definition	Example Quote
“Whiteness and Racism” 5 (10.20%)	White supremacy culture and divisions among white members of CTSAs and institutions	“2a White supremacy culture is deepened and BIPOC people and communities are further harmed”
“Structural vs Superficial Change” 6 (12.24%)	Large scale shift in the practices of CTSAs and their broader institutions. The need for large-scale change in order to achieve these priorities- change beyond the CTSA	“2A- Risk is that this will be isolated, rather than seen as paradigmatic for an institution, where these larger biases may also exist- so it might be an island, instead of the water.”
“New Perspectives” 7 (14.29%)	Expanding DEI in CTSA leadership could result in new, broader, more diverse perspectives	“2B impact: increased diversity in ideas, increased creativity, people might actually start listening to different voices and this would improve EVERYTHING. EVERYTHING. EVERYTHING.”
Priority 6: Recommendation E: Set a time limited goal (e.g. 90 days) to diversify the leadership, providing new leaders with the resources and authority to do the job they have been hired to do.		
“Theme” Tally “n” (% responses) Total *N* = 56	Definition	Example Quote
“Timeline” 33 (58.93%)	The concern that a quick timeline for meeting DEI goals is unsustainable	“5A. Very short timeframe and may lead people to be pressured to step into leadership before ready”
“Quotas” 6 (10.71%)	Numerical requirements for hiring based on race, ethnicity, gender, etc. and the controversy that sometimes surrounds this practice	“5a- Looks like a quota system”
“Qualifications” 7 (12.50%)	The experience, education, and other factors that deems a person prepared for a position	“5A. How do you ensure you are getting the best qualified not the one that fits the requirement”

## Methods

### Participants

Registrants of the Fall 2020 CTSA Consortium Meeting included: UL1 Principal Investigators (*n* = 30), Executive Director/Administrators (*n* = 35), KL2 Directors (*n* = 22), TL1 Directors (*n* = 15), CTSA Program Hub Steering Committee Members (*n* = 18), NCATS Program Officers or NIH Representatives (*n* = 7), and 117 participants held other positions. After an initial presentation on DEI efforts in translational science led by the meeting Chairperson, participants were asked to self-selectively join one of four breakout groups: leadership diversity, workforce development, research in health equity/disparities, and clinical trials recruitment. A total of 67 participants or 29% from the overall group were present in the leadership breakout session. The meeting was recorded and a consent clause was included in the online meeting registration. In addition, ethical and IRB procedures were followed at UNC. The data from the leadership breakout room are the focus of this analysis.

### Design

This study analyzes qualitative and quantitative data from transcripts of Zoom chats and Poll Everywhere results from 69 participants in the leadership breakout session in response to six recommendations (Table 1) aimed at advancing DEI efforts in CTSAs and the translational science field. For each recommendation, the facilitator asked one question about potential challenges and one question about potential opportunities presented by the possible implementation of each recommendation at CTSAs. After presenting the recommendations and allowing for participant commenting, they were then asked to upvote or downvote the recommendations according to feasibility, priority, and impact, using the anonymous Poll Everywhere web-based audience response system. The polls sequentially asked the participants to rank the recommendations according to: 1) *feasibility*, 2) *impact*, and 3) *priority*.

### Data Analysis

Two investigators independently coded the chat transcript data to look for themes by recommendation and cross-cutting themes across the recommendations in order to identify main challenges and opportunities. Using NVivo qualitative data analysis software, Coder One coded recommendations A–C and Coder Two coded recommendations D–F, creating a preliminary codebook. Each response was inductively coded and the tallies of each code were used to identify the most common themes by and across recommendations and priorities (Table 1). Both coders then compared and discussed codes to resolve discrepancies and create a finalized codebook with all six recommendations. The top three-to-five themes for each recommendation, as determined by tally count, and example quotes for each theme were recorded into the codebook.

Polling data was analyzed to identify the top recommendations by feasibility, impact, and priority using results from the three Poll Everywhere polls for each construct. Using the tallies from the NVivo analysis data, the two coders created a pyramid model to visualize the priority ranking of the six recommendations. Issue rankings from the three polls were averaged to determine a final aggregate ranking position and to create an overall model to depict participants’ attitudes towards the recommendations.

## Results

In response to recommendation A, “Identify a cross institutional P.I. action-learning workgroup…”, emerging themes included funding (*n* = 4 references, 8.89% of responses), cross-institutional (*n* = 5, 11.11% of references), new perspectives (*n* = 8 references, 17.78% of responses), and expansion (*n* = 9 references, 20.00% of responses). The primary barriers identified in implementing this theme were cross-institutional differences in culture, location, politics, etc., funding from senior leadership, and concern over whether this recommendation would limit individuals to solely P.I. roles. The primary opportunity identified in implementing this theme was new perspectives, meaning that DEI expansion in CTSA leadership could result in more diverse and novel ideas, projects, goals, etc.

In response to recommendation B, “Define a target of at least 30% of leaders…”, emerging themes included white privilege and racism (*n* = 5 references, 10.20% of responses), structural versus superficial change (*n* = 6 references, 12.24% of responses), and new perspectives (*n* = 7 references, 14.29% of responses). A common trend in the responses was the concern that setting a hiring target may be a superficial fix that ignores the broader, structural issues within the CTSA structure. White supremacy culture was also cited as a primary challenge in the potential implementation of this priority, while the theme of new perspectives was identified as the key opportunity this crucial issue presents.

In response to recommendation C, “Develop transparent policies, qualifications, applications and selection process for leadership roles,” emerging themes included institutional culture (*n* = 4 references, 9.09% of responses), institutional buy-in (*n* = 5 references, 11.36% of responses), and evaluation and monitoring (*n* = 7 references, 15.91% of responses). When asked about the feasibility of this recommendation, many respondents expressed excitement for the potential positive impacts this recommendation could lead to, yet recognized that institutional culture and buy-in could be barriers in the acceptance of changes to hiring processes for leadership roles, and emphasized the importance of comprehensive monitoring and evaluation strategies.

In response to recommendation D, “Define a clear succession plan…”, emerging themes included succession (*n* = 12 references, 25.53% of responses), resentment (*n* = 7 references, 14.89% of responses), and new perspectives (*n* = 8 references, 17.02% of responses). Resentment from non-URM individuals and/or individuals currently in power was identified as a primary barrier to successfully implementing this crucial issue. The theme of new perspectives emerged again as an opportunity that excited the participants, while the process of succession itself was discussed as both a barrier and an opportunity. Many participants expressed that succession planning can be a challenging task, but it allows for new leaders and ideas to emerge if done correctly.

In response to recommendation E, “Set a time limited goal (e.g. 90 day) to diversify the leadership,” emerging themes included timeline (*n* = 33 references, 58.93% of responses), quotas (*n* = 6 references, 10.71% of responses), qualifications (*n* = 7 references, 12.50% of responses), resources (*n* = 6 references, 10.71%), and evaluation and monitoring (*n* = 6 references, 10.71% of responses). The most pressing challenge identified was the concern that implementing a quick timeline for meeting DEI goals is unsustainable and could hinder progress. Additional discussion focused around quotas and qualifications, and if implementation of this recommendation would lead to hiring practices centered on meeting numerical requirements. Respondents also mentioned the need for appropriate resources, evaluation, and monitoring in order to achieve time-limited hiring goals.

In response to recommendation F, “Make room for structural change in DEI efforts,” emerging themes included new perspectives (*n* = 5 references, 11.36% of responses), feasible implementation (*n* = 5 references, 11.36% of responses), and stepping down (*n* = 5 references, 11.36% of responses). Feasible implementation emerged as both an opportunity and a barrier, depending on a CTSA’s individual practices, resources, and priorities. The theme of new perspectives was identified as the motivation to support implementing this recommendation. Many respondents also discussed the potential barrier of having individuals “give up” their position of power in order to achieve DEI efforts. Framing the recommendation in this way, according to participants, could cause controversy around this hiring practice.

Poll One (53 responses, 79% engagement) asked participants to upvote or downvote each of the six recommendations (A, B, C, D, E, and F) according to feasibility with the top three emerging as recommendation A (cross-Institutional P.I. workgroups), recommendation C (transparency), and recommendation D (clear succession plans), respectively.

Poll Two (58 responses, 88% engagement) asked participants to upvote or downvote each of the recommendations according to greatest impact with recommendation C (transparency), recommendation D (clear succession plans), and recommendation E (time-limited goals) as the top three, respectively.

Poll Three (59 responses, 88% engagement) asked participants to upvote or downvote each of the six recommendations according to which should receive the highest priority in implementation with recommendation C (transparency), recommendation A (cross-institutional P.I. workgroups), and recommendation D (clear succession plans) as the top three, respectively.

The outcome of the polling data is visualized in Fig. 1 and displays the overall ranking of the priority of recommendations A–F when scores were averaged across the three polls. The highest ranking priority across the three polls when voting was averaged is “Recommendation C: Develop transparent policies, qualifications, applications and selection process of leadership roles.” The lowest ranking priority across the three polls when voting was averaged is “Recommendation E: Set a limited goal (e.g. 90 day) to diversify the leadership, providing new leaders with the resources and authority to do the job they have been hired to do.”

## Discussion

The virtual technology-assisted discussion was energetic among the CTSA-workforce attendees, suggesting that the recommendations presented resonated with their lived experience and personal priorities at their places of employment. The prioritization process was multilayered to include perspectives of feasibility, impact, and priority for each recommendation, identifying the transparency of policies, qualifications, and the selection process for leadership roles as the highest priority, while their lowest priority was setting time-limited goals to diversify that leadership. There seemed to be a recognition, one shared across many industries, that substantial change in complex organizations comes slowly, and that setting a timeline, such as within a 90-day goal, was not a goal this group of CTSA members would prioritize, despite the polling data indicating a widely shared view that setting time-limited goals would have impact. Creating a peer group of leaders across organizations to facilitate co-learning provides networking and peer support was identified as a high priority. Not only is encouraging peer support groups a standard component of leadership development programs [11–13] but also this step is relatively easy to implement and could be facilitated by the CTSA network, even via the annual meeting. Peer support networks are effective when convened both in person and virtually, are inexpensive, require little training, and are highly flexible to meet the needs of the participants. A strong theme supporting the need for leadership development at CTSAs emerged from the prioritization process, with Priorities #2 and #3 both strongly benefitting from a focused and supported approach to build the skills and the supportive networks of leaders. Succession planning is greatly facilitated by developing the skills of a cadre of leaders in an organization, which helps mitigate the impact of turnover and benefits the leaders through the development of relationships that foster success at both learning and leading [14]. Nurturing leaders through intentional mentoring and structured action-learning/implementation science-focused projects has been shown to yield remarkable impact, even with small groups of individuals and small financial investments [12]. One such example of this approach is the Clinical Scholars program, which embraces an equity-centered leadership development model that has been shown to successfully develop peer networks and engage interprofessional and diverse teams to partner with communities to solve local challenging health equity problems [6,8,9,15–18]. The literature on DEI issues is an enthusiastic and rapidly growing area, yet such rapid expansion of ideas presents a challenge to “gold standard approaches” emerging. The outcomes of Clinical Scholars point to multiple strategies in equity-centered leadership development that could further benefit CTSAs in their future DEI goals. In terms of measuring DEI actions taken on the part of individuals, The Berkeley Rubric provides a model for assessing an individual’s knowledge of DEI and belonging, their actions to support that climate, and their actions towards advancing change in their institutions [19]. While the Berkeley Rubric does not provide the skills of a training program, it does suggest strategies for measurement of subsequent behavioral change and might be useful to those supporting DEI in CTSAs.

### Limitations

Given that the participants voluntarily registered for the meeting, the session, and self-selected to participate in the breakout session, they could represent a particular interest group and might not reflect the CTSA membership as a whole. Additionally, zoom identities are often the actual names of participants, and the lack of privacy could have impacted willingness to be transparent, although that impact is likely mitigated by the fact that participants were aware ahead of time that they would be interacting via chat. Such “waterfall” chats do not represent interactive discussion opportunities since participant input moves through the chat screen too quickly to assess and digest the contents. These findings call for a further assessment of the CTSA workforce on a much larger and anonymous scale to validate the extent to which these priorities are reflective of the wider workforce.

## Conclusions

While CTSAs serve as science incubators to benefit the public, their leaders and future leaders support structural changes that foster more equity in opportunities for leadership positions. They identified benefits from “incubating” and nurturing their potential through the implementation of organizational policies and practices. Several are relatively simple to institute and include transparency, cross-institutional action learning, and mentoring to support succession planning, all of which were rated as a high priority.
